# Load Monitoring Practice in Elite Women Association Football

**DOI:** 10.3389/fspor.2021.715122

**Published:** 2021-08-27

**Authors:** Live S. Luteberget, Kobe C. Houtmeyers, Jos Vanrenterghem, Arne Jaspers, Michel S. Brink, Werner F. Helsen

**Affiliations:** ^1^Department of Physical Performance, Norwegian School of Sport Sciences, Oslo, Norway; ^2^Department of Public Health, Sport and Nutrition, University of Agder, Kristiandsand, Norway; ^3^Faculty of Movement and Rehabilitation Sciences, Katholieke Universiteit Leuven, Leuven, Belgium; ^4^Center for Human Movement Sciences, University of Groningen, University Medical Center, Groningen, Netherlands

**Keywords:** training process, data analysis, physical training, team sport, female athletes, soccer

## Abstract

The description of current load monitoring practices may serve to highlight developmental needs for both the training ground, academia and related industries. While previous studies described these practices in elite men's football, no study has provided an overview of load monitoring practices in elite women's football. Given the clear organizational differences (i.e., professionalization and infrastructure) between men's and women's clubs, making inferences based on men's data is not appropriate. Therefore, this study aims to provide a first overview of the current load monitoring practices in elite women's football. Twenty-two elite European women's football clubs participated in a closed online survey (40% response rate). The survey consisted of 33 questions using multiple choice or Likert scales. The questions covered three topics; type of data collected and collection purpose, analysis methods, and staff member involvement. All 22 clubs collected data related to different load monitoring purposes, with 18 (82%), 21 (95%), and 22 (100%) clubs collecting external load, internal load, and training outcome data, respectively. Most respondents indicated that their club use training models and take into account multiple indicators to analyse and interpret the data. While sports-science staff members were most involved in the monitoring process, coaching, and sports-medicine staff members also contributed to the discussion of the data. Overall, the results of this study show that most elite women's clubs apply load monitoring practices extensively. Despite the organizational challenges compared to men's football, these observations indicate that women's clubs have a vested interest in load monitoring. We hope these findings encourage future developments within women's football.

## Introduction

Load monitoring in team sports has grown over the last decades (Bourdon et al., 2017). Load monitoring refers to the quantification, analysis and management of training load, and aims at refining the training process for improved training outcomes in terms of players' health and performance (Coutts et al., 2017; Impellizzeri et al., 2019). To understand current application, several studies have described the load monitoring practices in elite men's Association Football (hereafter called football) (Akenhead and Nassis, 2016; Weston, 2018; Houtmeyers et al., 2021). Insights from these studies can be used to stimulate further developments in both practice, academia and related industries such as technology providers. However, in contrast to elite men's football, no study has considered the load monitoring practices in elite women's football. Describing these practices would be valuable to gain specific insights in the developmental needs within women's football.

The lack of research focus on load monitoring in women's football may be explained by historical, cultural and financial reasons. Historically, there have been decades of differences between developments in men's and women's football; for example, the first FIFA World Cup for men was in 1930, while it took 61 years until women's football was included (1991). And 46 years passed between the first UEFA Champions League for men in 1955 and for women in 2001 (Williams, 2011). With limited attention to the women's game, financial resources were always only a fraction of the men's game at similar playing levels (FIFPro, 2020). In turn, this prevented professionalization and complementary advances in staff development. All-in-all, these differences certainly jeopardized the growth and development of women's football, yet nowadays it is clearly on the rise, indicated by a first fully professional women's league (WSL, England), increased stadium attendees, and a 10% increase in investments from national associations from 2017 to 2019 (UEFA, 2019). This accelerated growth and professionalization of the women's game has fueled research, especially in areas relating to sports medicine and strength and conditioning (Kryger et al., 2021). However, there is still a gap in the current research output between men's and women's football, with an average of 62% more publications per year on men's football compared with women's football over the last 10 years.^1^ Therefore, despite the newfound interest, many areas within women's football remain unexplored, such as load monitoring.

Monitoring of training load can help understand how players adapt to, and recover from training (Bourdon et al., 2017; Los Arcos et al., 2017). In addition, management of injury risk is shown as an important motivation to monitor training load (Akenhead and Nassis, 2016). These are important aspects of football, both for male and female players. Previous studies describing practices in men's football have shown that load monitoring is a part of the daily routine in many clubs (Akenhead and Nassis, 2016; Weston, 2018; Houtmeyers et al., 2021). However, the cost associated with monitoring of training load and the support staff available appears to be a limiting factor for implementation (Fox et al., 2020). Due to the obvious organizational differences (i.e., professionalization and infrastructure) that have existed, and still exist between the men's and women's game, problems arise when trying to make inferences based on men's data to generate insights on developmental needs for women's football. Data collected on men's football cannot simply be extrapolated to women's football without due considerations of the impact of that transfer. This encompasses both the challenges of comparing training load monitoring data due to physiological and anthropometric differences (Pedersen et al., 2019) (not in the scope of this study), and the practice of collecting and interpreting data due to organizational differences (i.e., professionalization and infrastructure) between men's and women's football clubs. Consequently, the description of current load monitoring practices in the context of women's football may serve to highlight challenges and to stimulate future development within women's football. Therefore, this study aims to outline an overview of the current load monitoring practices in women's football, by surveying a sample of elite European football clubs.

## Materials and Methods

### Participants

A total of 55 elite women's football clubs were invited to take part in a closed online survey (qualtrics.com). Clubs were invited if they competed in the top league of one of the 10 highest ranked European federations (UEFA Women's association club coefficients 2018/2019) and were represented by a single sports-science or sports-medicine staff member. These persons were approached via the national federation (*n* = 34), LinkedIN (*n* = 14) or the authors network (*n* = 7). Their participation was voluntary and was not stimulated by any incentive. All invitations were sent between January and September 2019. A first reminder was sent if there was no response within 1 month. A maximum of three reminders were sent if the survey remained unanswered. The study was conducted according to the requirements of the Declaration of Helsinki and was part of a research project that was approved by the KU Leuven ethics committee (s57732). All participants provided their consent after being informed about the purpose and procedures of the survey as well as the confidentiality and anonymity of the survey results.

### Design

The study consisted of a cross-sectional survey design. The content of the survey was similar to the one that was used in Houtmeyers et al. (2021). After being developed by the authors, the survey was evaluated by one researcher (Prof.) with expertise in survey design and construction. In addition, the face validity of the survey was evaluated by four applied researchers/practitioners with specific expertise in load monitoring [BSc. degree (*n* = 1), PhD. degree (*n* = 2), Prof. degree (*n* = 1); > 10 years of experience in physical training]. Based on these evaluations, four questions were modified, one question was added, and seven questions were deleted. The usability of the survey was pilot tested by two sports-science staff members of an elite youth academy (MSc. degree; ± 3 years of experience in physical training). The survey was available in six languages (Dutch, English, French, German, Italian and Spanish). Translation from English to the other five languages were made by researchers that were familiar with current terminology in load monitoring. Detailed information about the content of the survey can be found in the Supplementary Material. Questions were asked via multiple choice (*n* = 26) or a Likert scale (*n* = 7). Participants were able to change their answers via back buttons, were allowed to provide extra information in a text box and could respond with “no idea” if they did not know the answer. Five-point Likert scales were used and were fully labeled (1 = strongly disagree; 2 = somewhat disagree; 3 = neither agree nor disagree; 4 = somewhat agree; 5 = strongly agree). The survey was divided into three sections based on the topic of the questions. The first section included 13 multiple choice questions about the types of data collected and the collection purposes. In the second section, information was gathered about the applied analysis methods via seven Likert scale questions. The third and last section included 13 multiple choice questions about the involvement of the different types of staff members in the monitoring process.

### Statistical Analyses

Multiple choice and Likert scale questions were analyzed using frequency analysis and the results were presented as count (number of clubs) and the proportion of responded clubs, with single staff members representing their club.

## Results

The survey was completed by 22 clubs from 6 national federations [40% response rate (Table 1)]. Clubs were represented by sports-science (*n* = 20) and sports-medicine staff members (*n* = 2), having on average 5.26 ± 4.39 years of experience in football and 3.21 ±2.03 years of experience in their current club. Respondents were approached via the national federation (*n* = 10; response rate = 29%), LinkedIN (6; 43%) and the authors' network (6; 86%).

**Table 1:** Number of clubs invited per federation^*^ and corresponding response number and rate.

	**Invited**	**Responded**	**Rate**
France	3	3	100
Germany	6	3	50
Sweden	12	1	8
England	9	5	56
Spain	15	5	33
Italy	1	0	0
Netherlands	9	5	56
**Sum**	**55**	**22**	**40**

* *Ordered by the UEFA country coefficient 2018/19 (France, highest ranked; Netherlands, lowest ranked)*.

Of the 22 clubs, 18 (82%), 21 (95%), and 22 (100%) clubs collect external load, internal load and training outcome data, respectively. The number and proportion of clubs that collect external load, internal load and training outcome data for the different purposes of load monitoring is presented in Table 2. All (100%) of the clubs that collect external load data for training planning, also collect internal load data, while for the other applications this ranges between 69 and 92%.

**Table 2:** Number (proportion) of clubs that collect external load, internal load and training outcome data for the different purposes of load monitoring.

	**External load**	**Internal load**	**Training outcome**
Training planning	16 (73%)	21 (95%)	22 (100%)
Performance assessment	16 (73 %)	14 (64%)	21 (95%)
Game management	12 (55%)	13 (59%)	15 (68%)
Rehabilitation	13 (59%)	16 (73%)	21 (95%)
Youth development	8 (36%)	12 (55%)	13 (59%)

*A correspondence analysis of these result can be found in the Supplementary Materials*.

*External load: e.g. global navigation satellite system, accelerometer*.

*Internal load: e.g. heart rate monitor, rating of perceived exertion method*.

*Training outcome: e.g. aerobic fitness or neuromuscular fatigue assessment*.

**Figure 1:**
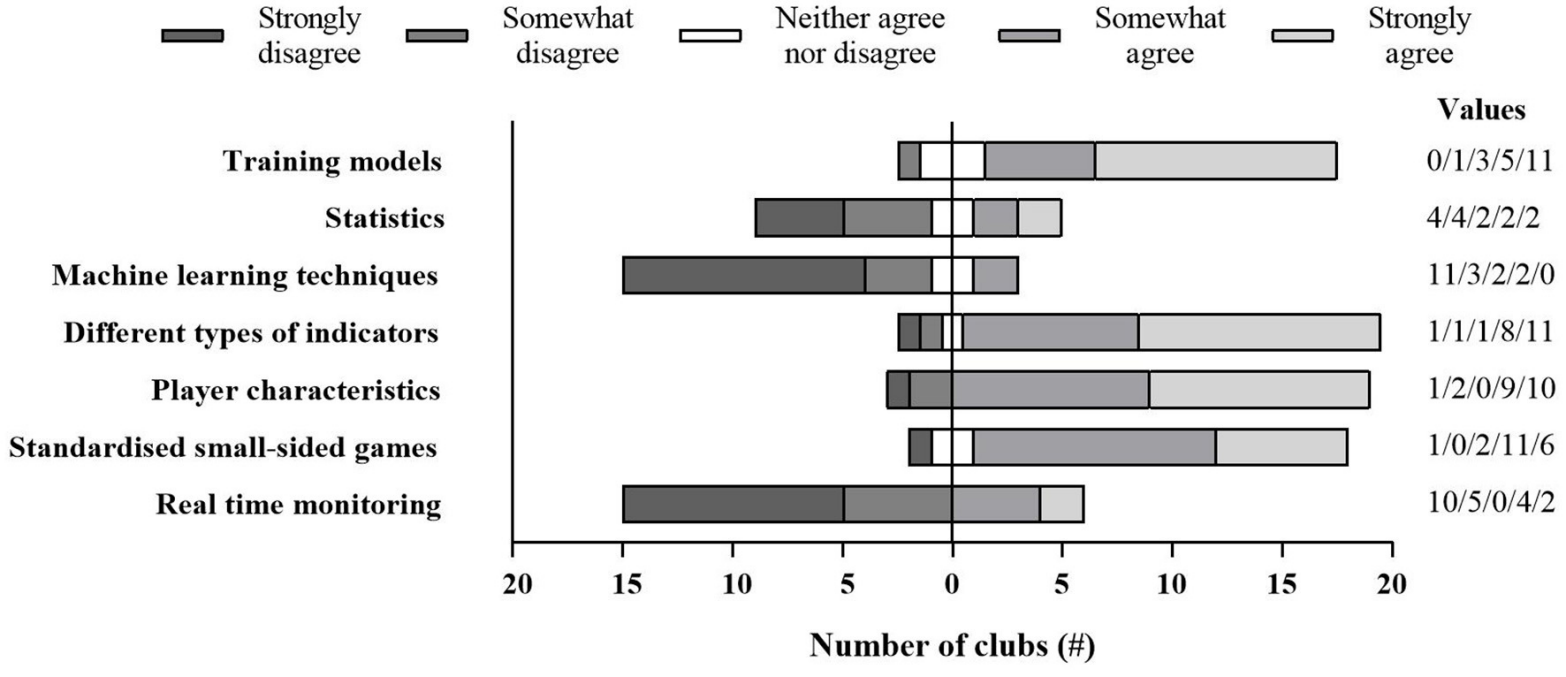
Response to the five Likert scale questions examining the load monitoring analysis methods. Results are presented as the number of clubs.

The majority of respondents indicated that their club use training models such as the acute-chronic workload ratio, fitness-fatigue model or monotony scores to analyse their data (Figure 1). Use of statistics, such as Z-scores, was indicated by 8 (36%) of the respondents, while only 2 (9%) indicate use of machine learning techniques. Furthermore, the majority of respondents indicated to take into account different types of indicators, combined with information on player characteristics (e.g., age, injury history), to make appropriate interpretations. Seventeen (77%) of the respondents indicated to use standardized small-sided games in function of the analysis, while 6 (27%) confirmed to make use of real-time monitoring during training or matches (Figure 1).

From one to ten staff member types were involved in each stage (Table 3) of the monitoring process. While the discussion stage involved the most types of staff members (mean ± SD: 6.1± 1.8), the data collection stage involved the least types of staff members (3.2 ± 1.7). The coaching staff is most often involved in data discussion and application. However, the goalkeeper coach is less involved than the head coach and assistant coach (Table 3). Sports-science staff members are involved throughout all stages, while sports-medicine staff members were mainly involved in data discussion. Presence of external staff members was low, except internship students who were present in 55% of the clubs.

**Table 3:** Presence and involvement of the different types of staff members in the monitoring process. Values are presented as the number (proportion) of clubs.

	**Presence**	**Involvement if present**				
		**Collection**	**Analysis**	**Reporting**	**Discussion**	**Application**
**Coaching staff**
Head coach	22 (100%)	2 (9%)	6 (27%)	4 (18%)	19 (86%)	17 (77%)
Assistant coach	22 (100%)	4 (18%)	5 (23%)	5 (23%)	18 (82%)	15 (68%)
Goalkeeper coach	22 (100%)	3 (14%)	4 (18%)	3 (14%)	11 (50%)	12 (55%)
**Sports-science staff**
Fitness or SandC^*^ coach	21 (95%)	18 (86%)	18 (86%)	19 (90%)	19 (90%)	18 (86%)
Performance manager	7 (32%)	3 (43%)	3 (43%)	4 (57%)	4 (57%)	4 (57%)
Sport scientist	9 (40%)	5 (56%)	5 (56%)	6 (67%)	8 (89%)	5 (56%)
**Sports-medicine staff**
Physiotherapist	22 (100%)	6 (9%)	9 (41%)	8 (36%)	18 (82%)	11 (50%)
Medical manager	14 (64%)	5 (36%)	8 (57%)	6 (43%)	8 (57%)	5 (36%)
Club doctor	19 (86%)	5 (26%)	6 (32%)	6 (32%)	10 (53%)	4 (21%)
**External staff**
University researcher	5 (22%)	2 (40%)	1 (20%)	1 (20%)	1 (20%)	1 (20%)
Internship students	12 (55%)	7 (58%)	3 (25%)	2 (17%)	3 (25%)	0 (0%)
Athlete management company	5 (22%)	2 (40%)	1 (20%)	2 (40%)	1 (20%)	2 (40%)
Self-employed consultant	2 (9%)	1 (50%)	1 (50%)	1 (50%)	1 (50%)	1 (50%)

*A correspondence analysis of these result can be found in the Supplementary Materials*.

* *SC,Strength and conditioning*.

## Discussion

This study aimed to provide an overview of the current load monitoring practices in elite European women football. While this has been investigated in men's clubs previously, to our knowledge, this is the first study to report on this from women's clubs. The results showed that all clubs (100%) collect data for training load monitoring purposes, and most clubs (82%) monitor several variables (external, internal, and training outcome) for several purposes. Training planning was the most frequently stated purpose for load monitoring, closely followed by performance assessment and rehabilitation. This is consistent with previous studies in men's football (Akenhead and Nassis, 2016; Weston, 2018; Houtmeyers et al., 2021). In addition, our data shows that multiple types of staff members are involved in the load monitoring process, and a range of analysis methods are being employed to interpret the data. This indicates that, despite the organizational challenges compared to men's football (i.e., professionalization and infrastructure), women's clubs have a vested interest in load monitoring.

In an applied environment, the ability to combine different load variables (external, internal, and training outcome) arguably offers a more ideal monitoring scenario than single load indicator schemes (Weaving et al., 2017). It has been emphasized in previous research that combined monitoring of external and internal load is necessary to gain insight into the association between load and training outcomes related to athletes' health and performance (Weaving et al., 2017; Impellizzeri et al., 2019). Our data suggest that load monitoring in women's football is implemented accordingly, where most clubs indicate that they collect multiple load variables for the different purposes. However, in contrast to previous studies in men's football (Akenhead and Nassis, 2016; Weston, 2018; Houtmeyers et al., 2021), internal load and training outcome data are collected in more clubs than external load data. This is particularly observable in the context of youth development and rehabilitation where external load data is the least used data source. This could be a consequence of the differences in financial resources between men's and women's football. External load monitoring often has a higher economical cost (Bourdon et al., 2017), both on the equipment but also on the staff side, as the amount of data collected increases the demands in terms of staff time and specialist expertise. In fact, its recently shown that greater financial resources facilitate extensive data collection in men's football (Houtmeyers et al., 2021).

The opportunity and ability to implement change and provide feedback based on data analysis is crucial for a successful monitoring system (Halson, 2014). Previous research in basketball have shown that training load data had minimal impact on modifications in the training plan (Fox et al., 2020), while in other sports, training load data support decision-making for athlete management (West et al., 2021). Although the degree to which implementation of changes based on the load monitoring occurred was not investigated in this study, the fact that a large number of staff was involved in the discussion and implementation phase of the load monitoring process is an indicator that the clubs view the load monitoring process as useful. In addition, analysis of the data can be considered an important but challenging aspect of the load monitoring process, especially when multiple data sources are present (Weaving et al., 2017). While several data sources can be important to establish a holistic view of the athletes, it also complicates the data analysis processes (Weaving et al., 2017). Our data show that a 86% of clubs in this study use multiple sources of data to make more appropriate interpretations. In addition, 86% of clubs indicate that they use player characteristics, such as age and injury history, and use standardized small-sided games (77%) in their load monitoring process. This shows that clubs use several approaches in the load monitoring, which can help the challenging process of interpreting trends in individual data (Ward et al., 2018). This could be especially important in women's football; while male players are full-time football players, many of their female peers have to work or study in addition to their daily training load as football players. Therefore, the contextual factors in women's football could possibly contribute to a larger degree to their total load.

Statistics such as the smallest worthwhile change and typical error of measurement allow a higher degree of confidence when interpreting observed changes and whether these changes should guide decisions about the training plan. Over half of the clubs in this study, to some extent, use statistics in their load monitoring analyses. However, what kind of statistics and to which extent they are used are still unknown. Training models are reported to be used by 77% of the clubs in this study in their analyses of load data. Training models have been highlighted as a practical and efficient method to calculate cumulative load and variations in load (Thornton et al., 2019). However, some training models, such as the acute-chronic workload ratio, have lately received attention due to inconsistent and controversial evidence (Impellizzeri et al., 2019; Dalen-Lorentsen et al., 2021). Since the data in this study were collected before these publications were available, the impact of these controversies in the scientific literature on the use of such models in the applied environment is currently unknown. The results of this study suggest that practitioners in women's football recognize the complexity of the load monitoring process and show that data analysis approaches are an integral part of the decision-making processes. Future research is needed to support these approaches with scientific and clinical evidence from the context of women's elite football, thereby focusing on the physiological difference between men and women (Randell et al., 2021).

This study provides evidence about the involvement of the different types of staff members in the monitoring process. Fitness or strength and conditioning coaches are the most involved in all stages of the monitoring process, and thus seem to have a more pronounced investment and responsibility in the process. This is also shown in research from men's clubs (Weston, 2018; Houtmeyers et al., 2021). Also, in line with previous research, the involvement of the coaching and the sports-medicine staff members was strong in especially the discussion and implementation stage of the load monitoring process. This indicates an interdisciplinary approach to decision-making, which is beneficial to exploit the collective expertise and experience from the different types of staff members. In fact, communication between different stakeholders is shown to be a key factor for increasing player availability (Ekstrand et al., 2019). However, this interdisciplinary approach also presents its challenges. For example, while coaches are expected to be skilled and experienced in their area, they are typically not experienced in data analysis or statistics (Robertson et al., 2017). This emphasizes the importance of effective and precise communication from staff members executing the analysis, ensuring that all stakeholders can interpret results for an effective discussion (Bartlett and Drust, 2020; West et al., 2021). This might be a particular challenge in women's football, as the current working environment often involves part-time employment of both staff and players. This administration of staff may present different challenges than in the men's football environments. In addition, the presence of external staff members was generally low, although over 50% of the clubs reported to have internship students present. If present, external staff were mostly involved in the data collection phase, which is in line with research in men's clubs (Houtmeyers et al., 2021). This might indicate that clubs need additional manpower to collect data in an efficient manner (Houtmeyers et al., 2021).

Providing insights into monitoring practices in women's football clubs, this study provides novel data that can further accelerate the knowledge and research interest into women's football. The study investigated high-competition level football clubs; however, a limitation of this study is that we used a convenience sample and did not approach all high-level clubs. In addition, survey studies have limitations, especially regarding response rates. This study had a response rate of 40%; a rate which is similar to previous research surveys involving football practitioners (Towlson et al., 2013; Mccall et al., 2014; Akenhead and Nassis, 2016; Gouttebarge et al., 2019; Houtmeyers et al., 2021). Although our survey was thoroughly evaluated, including feedback by a researcher with expertise in survey design and construction, we did not quantify the content validity of the survey. In addition, although the questions were translated into several languages, no cross-cultural validation was performed, which can be considered a limitation of the translation process. It is important to note that when invited to take part in this study, practitioners were informed about the topic of the survey. Therefore, we acknowledge the possible non-response bias in this study. This may have skewed our findings toward inflated load monitoring practices. Respondents from clubs in different countries and leagues strengthen our findings and decrease the likelihood of clustered response due to league or country specific practices and opinions. In this study, only closed ended questions were included. This gives more structured and interpretable data; however, we can miss some aspects of deeper insights in the load monitoring process. In future research, open ended questions or other methods such as qualitative interviews and focus groups could yield deeper insights into the practices of load monitoring (Kryger et al., 2021). In addition, this study describes the implementation of monitoring practices at a given time-point, with no information about the longitudinal perspective. The addition of such information in future research could yield insights into the continuity in the practice and amount of historical data available for analysis. Longitudinal study designs may also provide a greater understanding in how the different facilitators and barriers impact the implementation of load monitoring practices and might even provide specific insight into the effectiveness of interventions aimed at improving implementation. Although the current study focused on the organizational differences between men's and women's football, more detailed examinations may be valuable to better understand how physiological differences (e.g., menstrual cycle) between men and women impact or require specific load monitoring practices (Randell et al., 2021). Further, insights into the player perceptions of the load monitoring process and its outcomes could also yield insight into gaps from the practitioners to athletes' perspectives (Geertsema et al., 2021). In addition, inclusion of clubs on a lower level should be of interest in future research to investigate to which extent the current findings are similar across the different levels of competition in women's football.

## Conclusion

This study shows that most elite women's football clubs apply load monitoring practices extensively, using multiple data sources, including external load, internal load and training outcome data to inform their decision-making. Several analysis methods are used, including training models and standardized small-sided games. Fitness or strength and conditioning coaches are the staff members that seem to have the highest responsibility regarding load monitoring, however several staff members are involved in the load monitoring process, especially in the discussion stage. Collectively, these results indicate that, despite the organizational challenges compared to men's football (i.e., professionalization and infrastructure), elite women's clubs have a clear and obvious interest in load monitoring. The differences between men's and women's football warrant research underlining the specific challenges in these different environments. Particularly the dichotomy between resources and the intentions to monitor load may require careful considerations, both for researchers who support the development of the next generation(s) of load monitoring tools, and for practitioners. Therefore, the notion that women's clubs apply and prioritize the load monitoring process hopefully encourages future research into these practices in the context of women's football.

## Data Availability Statement

The raw data supporting the conclusions of this article will be made available by the authors, without undue reservation.

## Ethics Statement

The studies involving human participants were reviewed and approved by KU Leuven ethics committee (s57732 file number). The patients/participants provided their written informed consent to participate in this study.

## Author Contributions

KH, JV, AJ, MB, and WH conceptualized the study design and conducted the data acquisition. LL and KH contributed to the analysis of data and all authors contributed to the interpretation of the data. LL and KH drafted the manuscript and all other authors revised it critically. All authors approved the final version and agreed to be accountable for all aspects of this work.

## Conflict of Interest

The research grant contains a collaboration between the University of Leuven and the company TopSportsLab. The authors declare that the research was conducted in the absence of any commercial or financial relationships that could be construed as a potential conflict of interest.

## Publisher's Note

All claims expressed in this article are solely those of the authors and do not necessarily represent those of their affiliated organizations, or those of the publisher, the editors and the reviewers. Any product that may be evaluated in this article, or claim that may be made by its manufacturer, is not guaranteed or endorsed by the publisher.

## Footnotes

^1^These numbers are based on searches in PubMed. The search (football OR soccer) AND (females OR women OR ladies OR girls) was compared to (football OR soccer) AND (male OR men OR boys) in the time period from 2011 to 2020. Search words were based on search words in Kryger et al. (2021).
