# Supplementary material for: Load Monitoring Practice in Elite Women Association Football
Source: Front Sports Act Living. 2021 Aug 27;3:715122. doi: 10.3389/fspor.2021.715122 (PMC8430212; doi:10.3389/fspor.2021.715122)
Supplement: Supplementary file 1 [file Data_Sheet_1.PDF]

Supplementary Material – Survey design

1 Personal information

- Club
- Job Title
    - Sports-medicine staff member
    - Sports-science staff members
- Experience (years)
    - In football
    - In current club

2 Types of data collected and collection purposes (multiple-choice grid)

- Question
    - Please select the objectives and interventions for which you analyse player data using the following variables. You can select more than one variable per objective and intervention.
- Variables
    - External load (e.g. global navigation satellite system, local position measurement system, accelerometer)
    - Internal load (e.g. heart rate, lactate, rating of perceived exertion)
    - Training outcome (e.g. injury, fitness, neuromuscular fatigue, well-being)
- Objectives and interventions
    - Training planning
        - General planning (micro-, meso- and macrocycle)
        - Specific planning (passing drill, small-sided games, running drill)
        - Individual adjustment (overuse, detraining, underperformance)
        - Individual adjustment (physical development)
    - Performance assessment (team level)
        - Determination (evolution) team performance
        - Determination (evolution) player performance
        - Comparison performance between players
    - Game management
        - Selection starters, non-starters and reserves
        - Planning substitution strategy during the game
    - Rehabilitation
        - Planning return to sport process
        - Determination return to group training during rehabilitation
        - Determination return to competition during rehabilitation
    - Youth development
        - Physical preparation youth players for professional football demands

3 Applied analysis methods (Likert scale)

- Question
    - Please answer to the following statements
- Statements
    - Do you use
        - Training models (e.g. acute-chronic workload ratio, fitness-fatigue model, monotony)
        - Statistics (e.g. Z-scores, analysis of variance, magnitude-based inferences)
        - Machine learning techniques
        - Different types of indicators to make accurate interpretations

        - Player characteristics data to individualise the analysis
        - Standardised small-sided games
        - Real time-monitoring

4 Staff involvement (multiple choice grid)

- Question
    - Please select which role staff members/organisations have in the analysis process. You can select more than one role per staff member/organisation.
- Roles
    - Not present
    - No role
    - Data collection
    - Data analysis
    - Data reporting
    - Data discussion
    - Data application
- Staff members/organisations
    - Coaching department
        - Head coach
        - Assistant coach
        - Goalkeeper coach
    - Sports-science department
        - Fitness or S&C coach
        - Performance manager
        - Sport scientist
    - Sports-medicine department
        - Physiotherapist
        - Medical manager
        - Club doctor
    - External staff members
        - University researchers
        - Internship students
        - Athlete management company
        - Self-employed scientific consultant
