# Supplementary material for: Load Monitoring Practice in Elite Women Association Football
Source: Front Sports Act Living. 2021 Aug 27;3:715122. doi: 10.3389/fspor.2021.715122 (PMC8430212; doi:10.3389/fspor.2021.715122)
Supplement: Supplementary file 2 [file Data_Sheet_2.PDF]

Correspondence analysis

Figure 1 shows the relationship between the types of data collected and the collection purposes, which is presented in the article in table 2. Together, these results show that:

- Internal load and training outcome data are more often collected than external load data
- Internal load data are most often collected to plan training
- External load and training outcome data are the main source of information related to performance assessment
- Training outcome data are the main source of information related to rehabilitation
- External load data are less often collected in the context of youth development

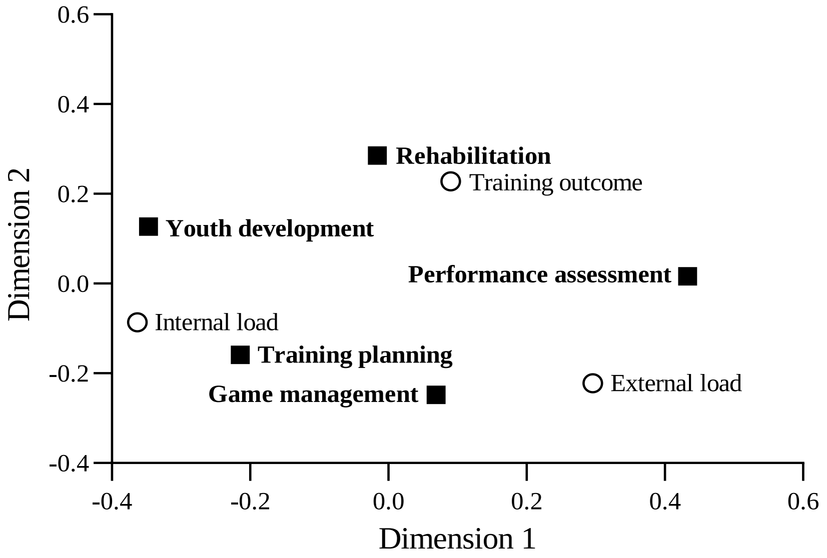

Figure 1: Correspondence analysis of types of data collected and the collection purposes. Analyses were performed using SPSS (version 26, IBM, New York, USA).

Figure 2 shows the relationship between the type of staff members and their involvement in the monitoring process (when they are present in the club), which is presented in the article in table 3. Together, these results show that:

- Coaching staff members are often involved in the discussion and application of load monitoring.
- Sport-science staff members are involved throughout all stages in the load monitoring process

- Sports-medicine staff members are mainly involved in data analysis and discussion
- If present, external staff members are mainly involved in data collection

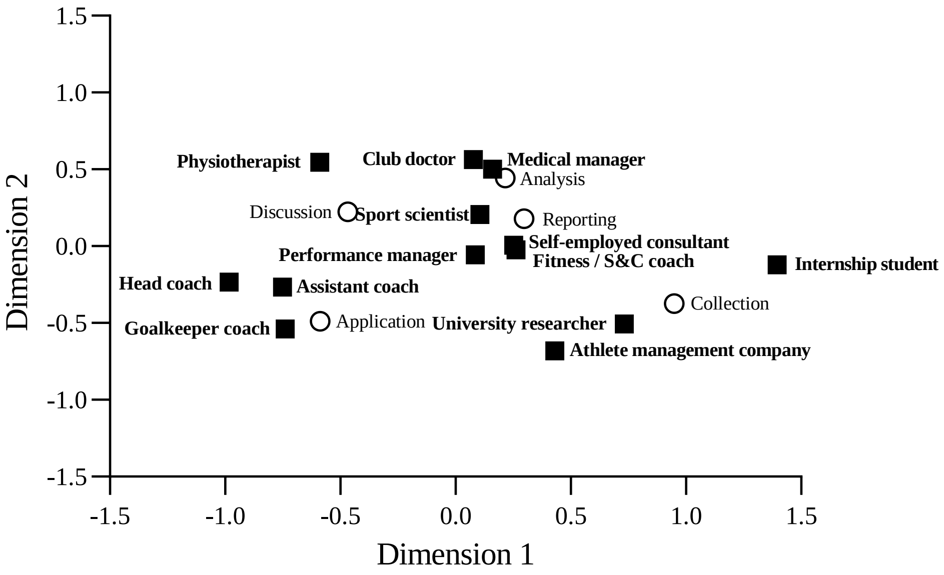

Figure 2: Correspondence analysis of type of staff members and their involvement in the monitoring process (when they are present in the club). Analyses were performed using SPSS (version 26, IBM, New York, USA).
